# Identification of a novel SXT/R391 integrative and conjugative element harboring *bla*_NDM-1_ in a clinical *Providencia huaxiensis* isolate

**DOI:** 10.3389/fmicb.2026.1818759

**Published:** 2026-05-22

**Authors:** Jia Tao, Wei Hong, Linyun Wang, Lixin Yan, Xinxin Hu, Jiale Li, Wei Jia, Yongxue Lv, Feng Li

**Affiliations:** 1 Center of Medical Laboratory, General Hospital of Ningxia Medical University, Yinchuan, China; 2 Ningxia Key Laboratory of Clinical and Pathogenic Microbiology, General Hospital of Ningxia Medical University, Yinchuan, China; 3 The First Clinical Medical College of Ningxia Medical University, Yinchuan, China; 4 School of Basic Medicine, Ningxia Medical University, Yinchuan, China

**Keywords:** *bla*_NDM-1_, *bla*_OXA-10_, carbapenem resistance, novel integrative and conjugative element (ICE), *Providencia huaxiensis*

## Abstract

In recent years, clinical *Providencia* species harboring carbapenemase-encoding genes have been increasingly reported, posing significant challenges in public health. However, *Providencia huaxiensis* (*P. huaxiensis*) isolates producing NDM have rarely been documented. Here, we report a carbapenem-resistant *Providencia* strain, PR66, which was initially misidentified as *P. rettgeri*. Antimicrobial susceptibility testing revealed resistance to all tested agents, including carbapenems. Species-level identification was confirmed using the average nucleotide identity (ANI) and digital DNA–DNA hybridization (dDDH). Whole-genome sequencing and bioinformatics analysis revealed that PR66 possesses a circular chromosome and three plasmids. The chromosome and plasmid pPR66.2 encode multiple resistance genes, including *bla*_NDM-1_, *bla*_OXA-4_, *bla*_OXA-10_, *bla*_MOX-3_, *aph(3’)-Ia*, *aph(3”)-Ib*, *aph(3’)-VIb*, *aph(6)-Id*, *aac(6’)-Ib3*, *ant(2”)-Ia*, *aadA*, *tet(B)*, among others. Notably, the *bla*_NDM-1_ gene is located on a novel integrative and conjugative element (ICE), ICE*Phu*Chn-PR66, which also harbors additional resistance genes. This ICE spanned 127,074 bp (positions 3,989,454–4,116,527 bp) and had a GC content of 48.6%. It was site-specifically integrated into the *prfC* locus of the PR66 chromosome. Conjugation and genetic stability assays confirmed the successful transfer of *bla*_NDM-1_ to *Escherichia coli* C600, with stable inheritance in the recipient strain. These findings underscore the role of ICEs in disseminating carbapenem resistance within *P. huaxiensis* and potentially across other bacterial species.

## 1 Introduction

The genus *Providencia* comprises Gram-negative bacteria within the order *Enterobacteriales* and family *Morganellaceae*. These organisms are widely distributed in various environments and are part of the normal gut flora of humans and animals (Wang et al., 2023). Members of this genus are implicated in diverse infections, particularly catheter-associated urinary tract infections, as well as pneumonia, endocarditis, sepsis, and meningitis.

The taxonomy of this genus has recently undergone substantial revision, expanding its phylogenetic depth. Based on latest update from the List of Prokaryotic names with Standing in Nomenclature (LPSN) (as of April 14, 2026), the genus *Providencia* comprises 18 validly published species names, including *P. rettgeri*, *P. stuartii*, *P. alcalifaciens* (Shaka et al., 2022), *P. rustigianii* (Hickman-Brenner et al., 1983), *P. heimbachae* (Mohr O'Hara et al., 1999), *P. burhodogranariea* (Juneja and Lazzaro, 2009), *P. sneebia* (Juneja and Lazzaro, 2009), *P. thailandensis* (Khunthongpan et al., 2013), *P. vermicola* (Somvanshi et al., 2006), *P. huaxiensis* (Hu et al., 2019), *P. hangzhouensis* (Dong et al., 2023), *P. manganoxydans* (Li et al., 2022), *P. xianensis* (Dong et al., 2024), *P. huashanensis* (Yang et al., 2024), *P. lanzhouensis* (Xiang et al., 2025), *P. xihuensis, P. zhejiangensis* (Dong et al., 2025), and *P. zhijiangensis* (Ksentini et al., 2019). Concurrently, genomic analyses have resolved several synonyms, clarifying that *P. thailandensis* is a later heterotypic synonym of *P. stuartii*, and *P. huashanensis* is a synonym of *P. xianensis* (Dong et al., 2025; Dong et al., 2024). Among all species, *P. rettgeri* is most frequently associated with human infections. Accurate species identification is essential for effective treatment. However, because of similarities in pan-genomic content and limitations of commercial biochemical identification systems, species such as *P. hangzhouensis* and *P. huaxiensis* are often misidentified (Dong et al., 2023). This challenge is exacerbated by the recent discovery of new species and the reclassification of existing ones. Average nucleotide identity (ANI) and digital DNA–DNA hybridization (dDDH) are widely accepted methods for species delineation, with established thresholds of 94–96% for ANI and 70% for dDDH.

Multidrug-resistant (MDR) *Providencia* strains, particularly those resistant to carbapenems, represent an escalating public health concern. Treatment is further complicated by their intrinsic resistance to colistin and tigecycline. *P. rettgeri* isolates carrying the carbapenemase genes have been reported globally, including in China (Peng et al., 2023), Colombia (Saavedra et al., 2022), Ecuador (Zurita et al., 2015), and Japan (Shiroto et al., 2005). These resistance genes can be horizontally transferred between bacterial cells mediated by mobile genetic elements (MGEs), mostly plasmids and integrative and conjugative elements (ICEs).

ICEs are a diverse group of mobile genetic elements that integrate into the bacterial chromosome and facilitate horizontal gene transfer (Botelho and Schulenburg, 2021). Among them, the SXT/R391 family of ICEs is widely distributed among Gammaproteobacteria, particularly within the order *Enterobacterales* (Han et al., 2024). These elements carry multiple resistance genes, including those conferring resistance to heavy metals, disinfectants, and clinically important antimicrobials such as extended-spectrum cephalosporins and carbapenems. SXT/R391 ICEs share a conserved backbone structure involved in integration, excision, and conjugation, while harboring variable regions that accommodate diverse resistance gene cassettes (Wozniak et al., 2009). Their ability to stably integrate into the bacterial chromosome and transfer across species makes them important vectors for the dissemination of antimicrobial resistance.

*P. huaxiensis* was first isolated from a human rectal swab in 2015 and formally described in 2019 in China (Hu et al., 2019). As a recently identified species, *P. huaxiensis* remains poorly characterized in terms of its antimicrobial resistance profile, molecular epidemiology, and the diversity of mobile genetic elements that may facilitate the horizontal transfer of resistance genes. In this study, we report a clinical NDM-producing *P. huaxiensis* isolate, PR66, which was primarily misidentified as *P. rettgeri*. Whole-genome sequencing (WGS) revealed a novel SXT/R391 ICE carrying *bla*_NDM-1_ in PR66, while *bla*_OXA-10_ was identified in chromosomal and plasmid associated integron contexts. This study aims to characterize the structural features, genetic context, and evolutionary relationships of this ICE, thereby elucidating the potential role of *P. huaxiensis* as a reservoir for carbapenem resistance genes.

## 2 Materials and methods

### 2.1 Species identification and antimicrobial susceptibility testing

Strain PR66 was isolated from the urine of a 51-year-old female patient admitted to a teaching hospital in the Ningxia Hui Autonomous Region, northwest China, for cerebral infarction treatment in 2017. The patient was admitted to the emergency ICU on April 17, presenting with dizziness, nausea, and vomiting, and was diagnosed with an acute cerebral infarction. The patient was transferred to the Neurological Critical Care Unit (NCU) on August 3 and subsequently to the Traditional Chinese Medicine ward for rehabilitation on August 25. Strain PR66 was obtained as a pure culture from a urine sample. Initial species identification of the strain PR66 was performed using matrix-assisted laser desorption/ionization time-of-flight mass spectrometry (MALDI-TOF MS) (BioMérieux, France). Antimicrobial susceptibility testing was performed using the VITEK 2 system (bioMérieux, France) following the Clinical and Laboratory Standards Institute (CLSI) guidelines. *Escherichia coli* ATCC 25922 and *Pseudomonas aeruginosa* ATCC 27853 were used as quality control strains. This study was approved by the Ethics Committee of the General Hospital of Ningxia Medical University (KYLL-2022-0689). All procedures were conducted in accordance with the relevant institutional and national guidelines.

### 2.2 Phenotypic and molecular detection of carbapenemase production

Phenotypic detection of carbapenemase activity was assessed using combined disc assays with imipenem alone and in combination with phenylboronic acid (PBA), EDTA, or both. This approach enabled the discrimination between metallo-B-lactamases (Class B carbapenemases, e.g., NDM) and KPC (Class A carbapenemases). Polymerase chain reaction (PCR) was performed to detect carbapenemase-encoding genes (*bla*_IMP_, *bla*_NDM_, *bla*_OXA-48_, and *bla*_KPC_). Primers were designed using Primer Premier 5.0 program (Singh et al., 1998). Positive amplicons were subjected to Sanger sequencing (Sangan Company, Shanxi, China), and the sequence data were analyzed using the BLAST tool.^1^ Primer sequences used in this study are listed in Supplementary Table 1.

### 2.3 Sequencing and assembly

Genomic DNA of PR66 strain was isolated using TGuide S96 Magnetic Universal DNA Kit (TianGen, China). Illumina libraries were prepared from 1 ng of gDNA using the VAHTSTM Universal Plus DNA Library Prep Kit and sequenced on a NovaSeq6000 platform (Illumina Inc., San Diego, CA, United States) with 2 × 150-bp paired-end reads. For PacBio sequencing, a SMRTbell library was prepared from gDNA sheared to 15–20 kb and sequenced on a PacBio HIFI platform (Pacific Biosciences, Menlo Park, CA, United States), generating approximately 200 × coverage with a mean read length of 12 kb. The completed genome sequence was assembled using Hifiasm v0.12 software (Cheng et al., 2021). The genome was circularized, and the starting position was adjusted with Circlator v1.5.5 (Hunt et al., 2015), and further polished with Pilon v1.22 (Walker et al., 2014) using Illumina sequencing data to obtain a higher-accuracy genome.

### 2.4 Precise identification

For precise taxonomic classification, 78 *Providencia* genus genome sequences were obtained from the National Center for Biotechnology Information (NCBI) database. PyANI v0.2.13^2^ and GGDC v3.0 (Emms and Kelly, 2019) were used to calculate ANI and dDDH values between the PR66 genome and the 78 *Providencia* genus genome of *Providencia* genus.

### 2.5 Phylogenetic analysis of *Providencia* spp.

Orthologous gene analysis was performed using OrthoFinder v2.4.0 (Castresana, 2000) with the default parameters. Multiple sequence alignments of each identified single-copy ortholog were generated using MUSCLE v3.8.31.^3^ Low-quality terminal alignments were excluded using the GBLOCKS v0.91b (Letunic and Bork, 2021). A custom script was used to concatenate the aligned sequences in a uniform order. Phylogenetic relationships were inferred using FastTree v2.1.11^4^ based on the Maximum Likelihood (ML) method (bootstrap test with 1,000 replicates), which was visualized using iTOL v7 (Hyatt et al., 2010).

### 2.6 Sequence annotation and comparison

Protein coding genes were predicted using Prodigal v2.6.3 (Carattoli and Hasman, 2020). Genomic sequences were annotated using NCBI non-redundant protein database (NR) (2024-02-7). PlasmidFinder v4.1 (Bortolaia et al., 2020) and ResFinder v4.0 (Liu et al., 2019) were used to identify plasmid replicons and resistance genes, respectively. ICE analysis was conducted using ICEberg v0.3.0 (Alikhan et al., 2011). Genomic comparisons and visualizations were performed using BRIG v0.95 (Sullivan et al., 2011) and EasyFig v2.2.2 (Ondov et al., 2016).

### 2.7 Phylogenetic analysis of the SXT/R391 ICEs

Phylogenetic analysis of the SXT/R391 ICEs was performed to investigate the evolutionary origins of the SXT/R391 ICE in the PR66 strain. The sequence of ICE*Phu*Chn-PR66 was subjected to BLAST alignment against the NCBI database to obtain closely related ICEs in the public database. The distance matrix between sequences was calculated using the MASH v2.1 software (Liu et al., 2023). Subsequently, clustering was performed using the Hcluster package in R based on the average linkage method to generate a phylogenetic tree, which was then visualized using iTOL v7 (Hyatt et al., 2010).

### 2.8 Conjugative transfer of *bla*_NDM-1_ positive ICE

Conjugation experiments were conducted to assess the transferability of the *bla*_NDM-1_-positive ICE. *P. huaxiensis* PR66 was used as the donor strain, and rifampin-resistant *E. coli* C600 was chosen as the recipient. Both strains were cultured to the exponential growth phase (reaching an optical density of approximately 0.5 at 600 nm) and combined at a donor-to-recipient ratio of 1:1. After 24 h of incubation at 37 °C, transconjugants were selected on MacConkey agar supplemented with rifampicin (100 mg/L) and meropenem (4 mg/L). Transconjugants were identified using MALDI-TOF MS and confirmed by PCR. Antimicrobial susceptibility profiles were determined using the VITEK-2 system.

### 2.9 Genetic stability testing of *bla*_NDM-1_ positive ICE

The genetic stability of the transferred ICE was evaluated by serial passage of *E. coli* C600 transconjugants (E600/PR66) in antibiotic-free LB medium. Cultures were diluted 1:1000 daily and incubated at 37 °C with shaking at 220 rpm for 10 consecutive days (approximately 100 generations). PCR was performed daily to monitor the presence of *bla*_NDM-1_ genes.

## 3 Results

### 3.1 PR66 strain information

PR66 was initially identified as *P. rettgeri* using MALDI-TOF MS. The isolate exhibited resistance to all tested antimicrobials, including cefepime, ceftazidime, ceftriaxone, aztreonam, piperacillin-tazobactam, ampicillin-sulbactam, ticarcillin-clavulanate, meropenem, imipenem, amikacin, tobramycin, ciprofloxacin, and levofloxacin (Table 1). Phenotypic assays confirmed metallo-*β*-lactamase (MBL) production, and PCR followd by Sanger sequencing confirmed of the presence of the *bla*_NDM-1_ gene (Supplementary Figure S1). Whole-genome analysis revealed that PR66 harbored a circular chromosome of 4,611,641 bp and three plasmids: pPR66.1 (206,981 bp), pPR66.2 (145,112 bp), and pPR66.3 (7,081 bp). The GC contents of the chromosome and plasmids were 41, 45.4, 47.1, and 32.3%, respectively. ResFinder server analysis identified multiple resistance genes, including *β*-lactam resistance genes (*bla*_NDM-1_, *bla*_OXA-4_, *bla*_OXA-10_, *bla*_MOX-3_), aminoglycoside resistance genes (*aph(3′)-Ia*, *aph(3″)-Ib*, *aph(3′)-VIb*, *aph(6)-Id*, *aac(6′)-Ib3*, *ant(2″)-Ia*, *aadA*), and the tetracycline resistance gene (*tet(B)*), among others. These findings were consistent with the phenotypic resistance profiles. Notably, *bla*_OXA-10_ was found on both the chromosome and the plasmid pPR66.2. The resistance genes are listed in Table 2. Three prophage regions were identified: two on the chromosome and one on plasmid pPR66.2.

**Table 1:** Antimicrobial susceptibility of the clinical *P. huaxiensis* PR66 strain and transconjugant.

Antimicrobial class	Antimicrobial agents	MIC(μg/mL)		*E. coli* C600
		PR66	EC600/PR66	
Cephalosporins	Cefepime	≥64 (R)	≥64 (R)	≤1 (S)
	Ceftazidime	≥64 (R)	≥64 (R)	≤1 (S)
	Ceftriaxone	≥64 (R)	≥64 (R)	≤1 (S)
*β*-lactam/*β*-lactamase inhibitor combinations	Ticarcillin-clavulanate	≥128 (R)	≥128 (R)	≤8 (S)
	Ampicillin-sulbactam	≥32 (R)	≥32 (R)	4 (S)
	Piperacillin-tazobactam	≥128 (R)	≥128 (R)	≤4 (S)
Monobactams	Aztreonam	≥64 (R)	32 (R)	≤1 (S)
Carbapenems	Imipenem	≥16 (R)	≥16 (R)	≤0.25 (S)
	Meropenem	≥16 (R)	≥16 (R)	≤0.25 (S)
Aminoglycosides	Tobramycin	≥8 (R)	≥16 (R)	≤1 (S)
	Amikacin	≥64 (R)	4 (S)	≤2 (S)
Fluoroquinolones	Levofloxacin	≥8 (R)	0.5 (S)	0.5 (S)
	Ciprofloxacin	≥4 (R)	0.5 (S)	≤0.25 (S)
Sulfanilamides	Trimethoprim/sulfamethoxazole	≥16 (R)	≥16 (R)	≤1 (S)

S, susceptible; I, intermediate; R, resistant.

**Table 2:** Antibiotic resistance genes in *Providencia huaxiensis* PR66 genome.

Location	Antimicrobial agents	Resistance genes	Identity (%)	Alignment length/gene length	Start	End
Chromosome of PR66 strain	Beta-lactam	*bla*_NDM-1_	100	813/813	4,075,542	4,076,354
		*bla*_OXA-4_	100	831/831	4,081,453	4,082,283
		*bla*_MOX-3_	99.13	1149/1149	4,089,303	4,090,445
		*bla*_OXA-10_	100	801/801	4,094,929	4,095,729
	Aminoglycoside	*aph(3′)-Ia*	100	816/816	531,371	532,186
			100	816/816	529,297	530,112
		*aph(6)-Id*	100	837/837	509,388	510,224
		*aph(3″)-Ib*	100	803/803	508,585	509,388
		*aph(3′)-VIb*	99.23	778/780	4,069,332	4,070,109
		*aac(6′)-Ib3*	99.82	555/555	4,082,414	4,082,968
		*ant(2″)-Ia*	100	534/534	4,096,511	4,097,044
		*aadA*	99.87	792/792	4,094,121	4,094,912
	Rifampicin	*arr-3*	100	453/453	4,074,349	4,074,801
	Phenicol antibiotic	*floR*	98.35	1214/1215	518,852	520,065
		*catB3*	100	633/633	4,080,683	4,081,315
		*catB8*	97.16	633/633	4,095,798	4,096,430
	Sulfonamide antibiotic	*sul2*	100	816/816	522,569	523,384
		*sul1*	100	840/840	4,092,777	4,093,616
			100	840/840	4,079,346	4,080,185
			100	840/840	4,072,946	4,073,785
	Diaminopyrimidine antibiotic	*dfrA1*	100	474/474	38,428	38,901
Plasmid pPR66.2	Beta-lactam	*bla*_OXA-10_	100	801/801	68,302	69,102
	Aminoglycoside	*aadA1*	99.87	792/792	67,494	68,285
		*ant(2″)-Ia*	100	534/534	69,884	70,417
	Phenicol	*catB8*	97.16	633/633	69,171	69,803
	Sulfonamide	*sul1*	100	840/840	66,150	66,989
	Tetracycline	*tet(B)*	100	1206/1206	57,621	58,826

### 3.2 Precise identification and phylogenetic analysis of *Providencia* strains

**Figure 1:**
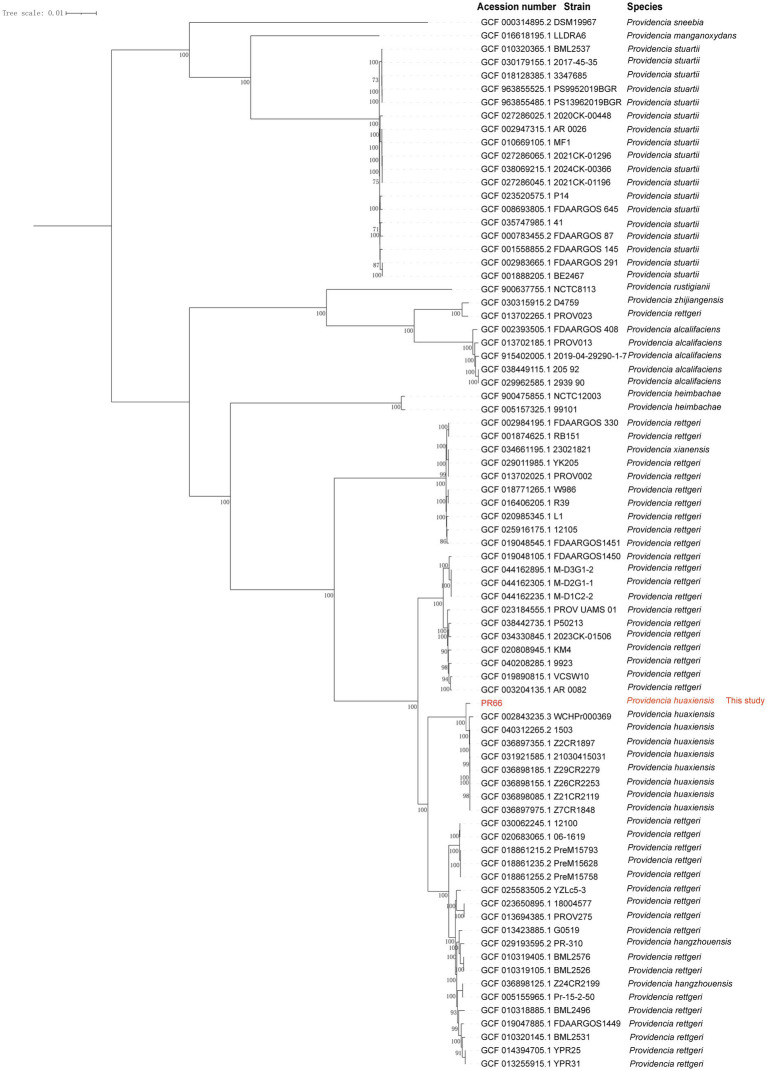
Maximum-likelihood phylogenetic tree inferred from 1,375 single-copy orthogroups from 79 *Providencia* strains. The tree defines the phylogenetic position of PR66 and clarifies species-level clustering after re-evaluation of the downloaded genomes. Of the 78 genomes retrieved from public databases, 27 originally annotated as *P. rettgeri* were reassigned, including 17 to *P. hangzhouensis*, 9 to *P. xianensis*, and 1 to *P. zhijiangensis*. Numbers at the nodes indicate bootstrap support values from 1,000 replicates.

ANI and dDDH analyses revealed that PR66 shared 98.93% ANI and 91.2% dDDH with *Providencia huaxiensis* WCHPr000369 (GCF_002843235.3), confirming its identity as *P. huaxiensis.* Further analysis revealed taxonomic inconsistencies among the genomes of *Providencia* spp. in the NCBI database. A total of 78 *Providencia* spp. genomes were downloaded (Supplementary Table 2), among which 27 strains labeled as *P. rettgeri* were misclassified. Re-identification showed that most were *P. hangzhouensis* (*n* = 17), *P. xianensis* (*n* = 9), and *P. zhijiangensis* (*n* = 1). Heatmaps of the pairwise ANI and dDDH values for the 79 *Providencia* strains are shown in Supplementary Figures S2, S3, with detailed values in Supplementary Tables 3, 4. A total of 8,290 gene clusters were identified, reflecting the high genetic diversity. Among these, 1,505 orthologs (18.2%) constituted the core genome, whereas 6,735 (81.3%) and 49 (0.6%) were classified as accessory and species-specific genes, respectively. A set of 1,375 single-copy orthogroups was used to construct a phylogenetic tree (Figure 1), which showed accurate clustering of strains by species.

### 3.3 A novel SXT/R391 family ICE ICE*Phu*Chn-PR66 harboring the *bla*_NDM-1_ gene on the chromosome

**Figure 2:**
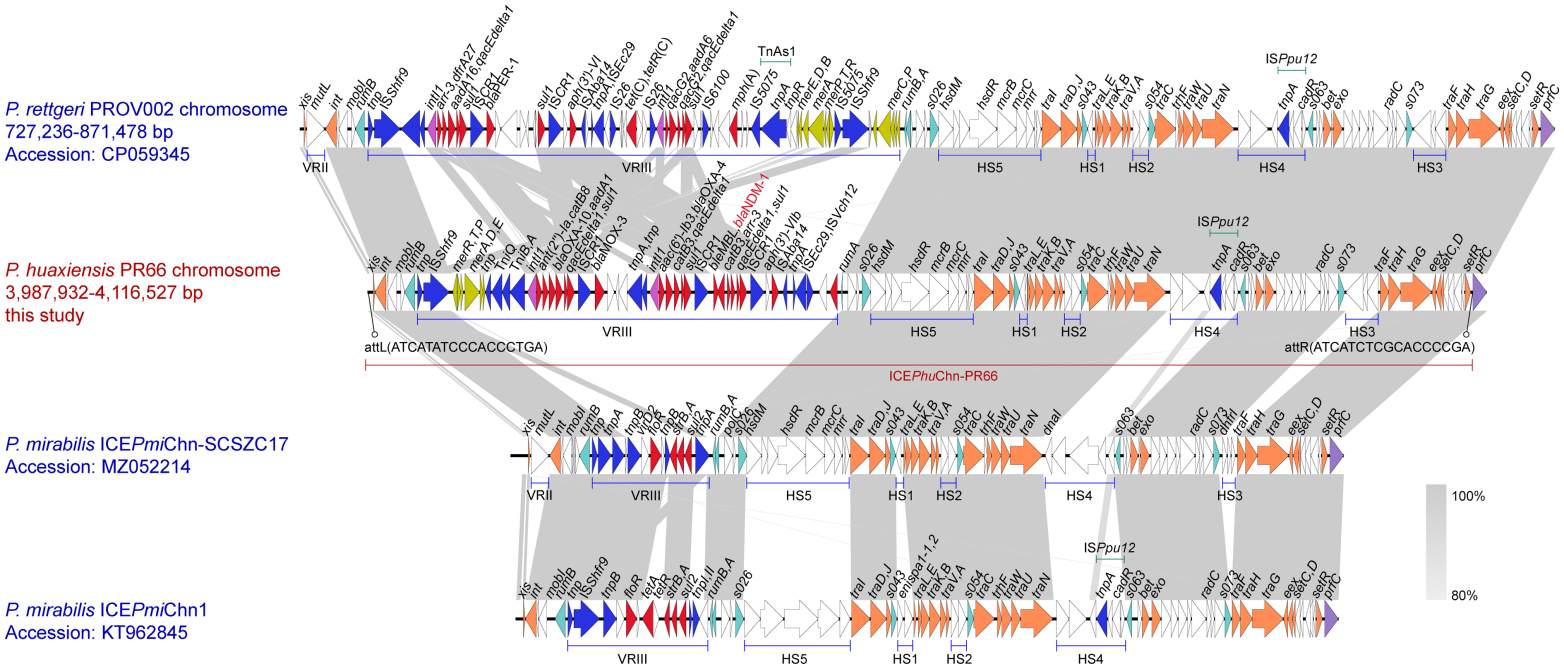
Comparative analysis of the structures of ICE*Phu*Chn-PR66 from PR66 strain and related SXT/R391 family integrative and conjugative elements (ICEs). The comparison illustrates the conserved SXT/R391 backbone of ICE*Phu*Chn-PR66 while delineating differences in accessory regions relative to related elements. ICE*Phu*Chn-PR66 is a 127,074-bp chromosomal element inserted at the *prfC* locus and contains recombination, conjugation, regulatory, and multidrug-resistance regions, including VRIII and five hotspot insertion sites (HS1–HS5). Orange denotes ICE backbone genes; light blue denotes genes adjacent to variable regions (VRs) or hotspot sites (HSs); dark blue denotes insertion sequences; pink denotes integrase; red denotes antimicrobial resistance genes; lime green denotes heavy metal resistance genes; and purple denotes genes at the chromosomal insertion site. Red-highlighted regions emphasize resistance cargo, whereas orange highlights the conserved backbone shared by the compared ICEs.

ICEfinder prediction and BLAST comparison revealed an ICE of 127,074 bp (positions 3,989,454–4,116,527 bp) was site-specifically integrated into the *prfC* locus of the PR66 chromosome, with a GC content of 48.6%. In addition to *bla*_NDM-1_ gene, this ICE region also carries other antimicrobial resistance genes, including *bla*_OXA-4,_ *bla*_MOX-3,_ *bla*_OXA-10,_ *aph(3′)-VIb, aac(6′)-Ib3, ant(2″)-Ia, aadA, arr-3, catB3, catB8, sul1.* Using the ICE*Pmi*Chn1 sequence (GenBank accession number: KT962845) as the reference sequence, we found this ICE element, designated ICE*Phu*Chn-PR66, exhibited a highly conserved backbone structure characteristic of the SXT/R391 family of ICEs (Figure 2). ICE*Phu*Chn-PR66 includes recombination, conjugation, regulation, and MDR regions, including VRIII and five hotspot insertions (HS1–HS5). BLAST comparisons revealed structural similarity to ICE on the *P. rettgeri* PROV002 chromosome (CP059345.1), with identical insertion sequences in HS1–HS5. Differences were observed in the VRIII MDR region and the presence of a mutL gene insertion in the VRII region of PROV002. Similar ICE structures have also been found in *Providencia* spp. PROV003 chromosome (CP096369.1) and *P. rettgeri* YK205 chromosome (CP090217.1), suggesting a possible common origin for these ICEs. Comparative analysis using BLASTn against known ICE sequences revealed two related elements: *P. mirabilis* ICE*Pmi*Chn-SCSZC17 (MZ052214.1) and ICE*Pmi*Chn1 (KT962845.1). ICE*Pmi*Chn-SCSZC17 shares insertion genes in HS1, HS2, and HS5 but differs in HS3, HS4, and VRIII. The former also has a mutL gene insertion in the VRII region, similar to that of PROV002. ICE*Pmi*Chn1 differs in HS1, HS2, HS5, and VRIII but shares HS4 insertions. No ICEs identical to ICE*Phu*Chn-PR66 were found, suggesting that it is a novel MDR SXT/R391 ICE capable of horizontal transfer of carbapenem resistance.

### 3.4 Phylogenetic analysis of SXT/R391 ICEs

**Figure 3:**
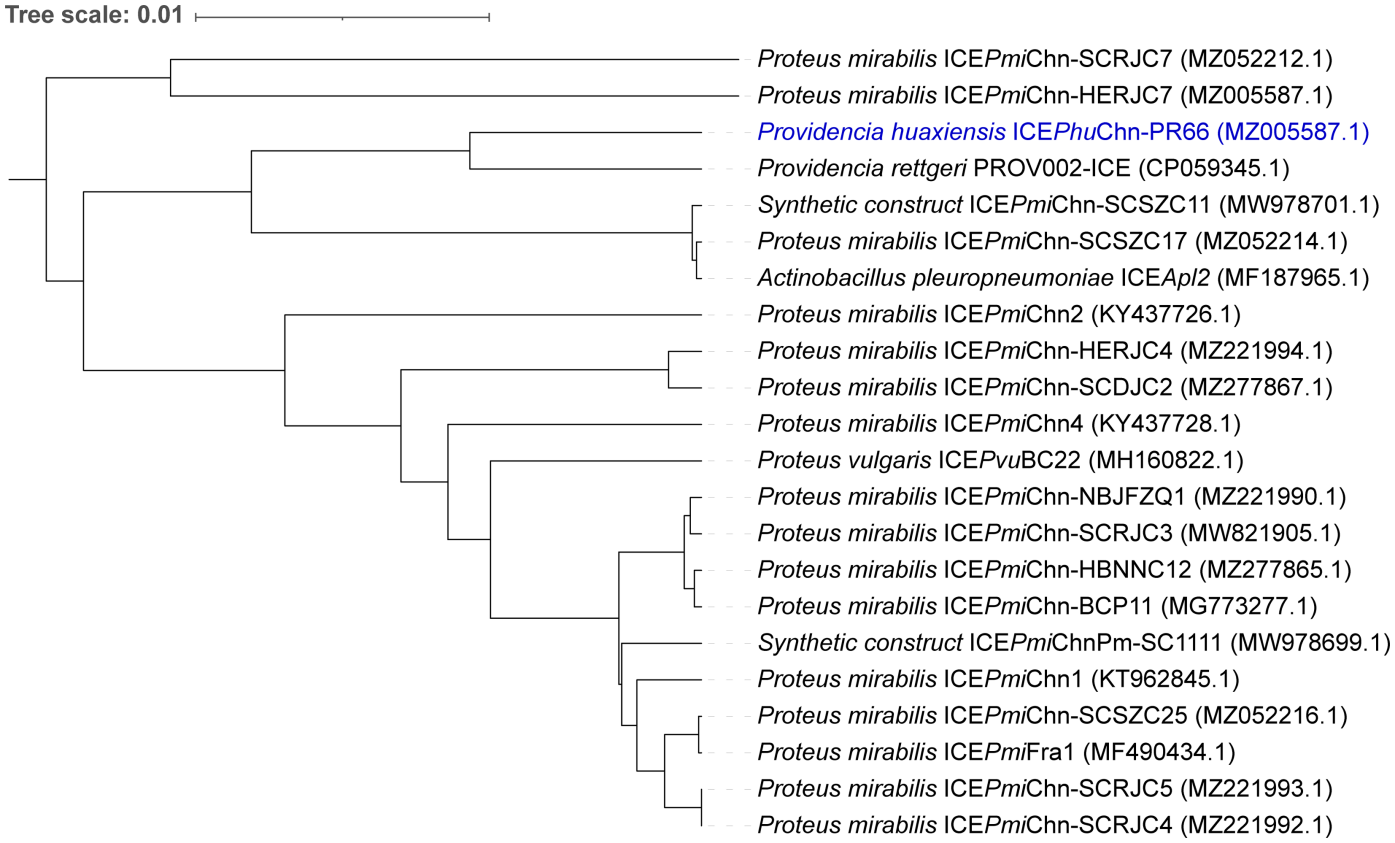
Phylogenetic tree based on core genome alignments of the SXT/R391 ICEs. ICE*Phu*Chn-PR66 was compared with 21 reference SXT/R391 ICE sequences retrieved from public databases. The scale bar indicates substitutions per site.

Blastn results showed that ICE*Phu*Chn-PR66 had query coverage ranging from 43 to 100% and identity ranging from 96 to 100% compared to other SXT/R391 ICEs in the NCBI database. A total of 21 reference sequences were obtained for ICE evolutionary analysis (Supplementary Table 5). These ICEs were derived from *Actinobacillus pleuropneumoniae*, *Proteus mirabilis*, *Proteus vulgaris*, *Synthetic construct*, and *Providencia rettgeri*. The phylogenetic analysis revealed that the ICE*Phu*Chn-PR66 clustered closely with PROV002-ICE from *P. rettgeri* and formed a distinct clade separate from the majority of *Proteus mirabilis* ICE elements. Notably, the *P. huaxiensis* ICE*Phu*Chn-PR66 shared a more recent common ancestor with *P. rettgeri* PROV002-ICE than with most *P. mirabilis* isolates, suggesting potential interspecies horizontal transfer of ICE elements between *Providencia* and *Proteus* species (Figure 3).

### 3.5 A pCHS4.1-3-like plasmid pPR66.2 harboring *bla*_OXA-10_

**Figure 4:**
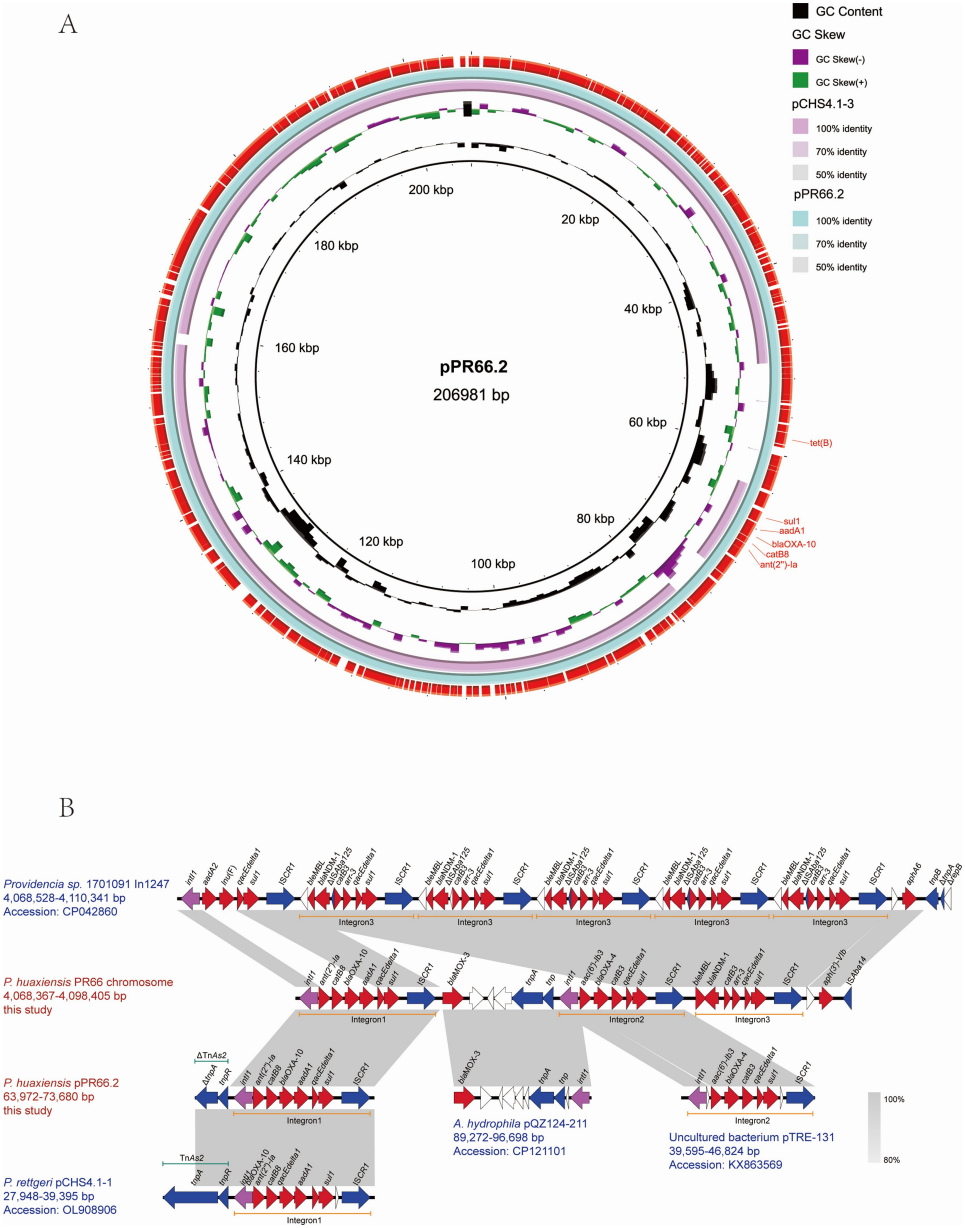
Comparative analysis of the *bla*_OXA-10_-associated plasmid and integron structures in PR66 strain. **(A)** Comparison of plasmid pPR66.2 with other plasmids. The circles show (from inside to outside): scale in 5 kb, GC content, GC skew [(G–C)/(G + C)], and predicted ARGs of pPR66.2 plasmid. **(B)** Comparison of the *bla*_OXA-10_ carrying integrons. Genes are represented by arrows. Red represents resistance genes, light purple represents integrase, and blue represents insertion sequence.

Antimicrobial resistance genes prediction showed *bla*_OXA-10_ was found on both the chromosome and the plasmid pPR66.2. Plasmid pPR66.2 and the *bla*_OXA-10_ gene environment were also analyzed to compare the genomic environments of the two *bla*_OXA-10_ gene copies. PlasmidFinder analysis indicated that pPR66.2 did not match any known plasmid types but shared 99.89% identity with plasmid pCHS4.1 (OL908908) from the clinical *P. rettgeri* strain CHS4 in China, based on BLASTN analysis (Figure 4A). The *bla*_OXA-10_ gene was located within the integrons present on both the chromosome and plasmid pPR66.2 (Figure 4B). The genetic environment of *bla*_OXA-10_ gene was int*1*-*ant(2″)-Ia*-*catB8*-*bla*_OXA-10_-*aadA1*-*qacEdelta1*-*sul1*-ISCR*1*, which was similar to the plasmid pCHS4.1-1 (OL908906) in *P. rettgeri*.

### 3.6 Conjugation experiments and conjugate stability

Conjugation assays confirmed the successful transfer of *bla*_NDM-1_ into *E. coli* C600. Transconjugants carrying the *bla*_NDM-1_ gene were confirmed using PCR. Susceptibility testing showed that the transconjugants exhibited resistance to carbapenems (imipenem and meropenem), cephalosporins (cefepime, ceftriaxone, and ceftazidime), and *β*-lactam/*β*-lactamase inhibitors (piperacillin-tazobactam) (Table 1), consistent with the resistance phenotype observed in the parental strains (Table 2). Stability testing showed that *bla*_NDM-1_ gene was retained in transconjugants after 10 d of culture in LB broth without antibiotic selection. Daily PCR confirmed the presence of the *bla*_NDM-1_ gene (Supplementary Figure S4).

## 4 Discussion

In this study, we identified the clinical *P. huaxiensis* strain PR66 using ANI and dDDH comparisons. The strain was initially misidentified as *P. rettgeri* by MALDI-TOF MS. Similarly, we observed the misidentification of several *Providencia* genomes in the NCBI database, many of which were incorrectly labeled as *P. rettgeri*. Precise reclassification revealed that most of these strains were *P. hangzhouensis*, followed by *P. xianensis* and *P. zhijiangensis*. Dong *et al*. previously reported that *P. huaxiensis*, *P. hangzhouensis,* and *P. rettgeri* share similar pan-genomic features, underscoring the need for accurate species identification within the *Providencia* genus. Accurate identification of bacterial pathogens is essential for effective clinical management and epidemiological surveillance of infectious diseases. Misidentification can result in inappropriate antibiotic selection, leading to poor patient outcomes, prolonged hospitalization, and increased mortality rates. Furthermore, evolutionary studies depend on reliable genomic data, and misannotated sequences in public databases can distort phylogenetic relationships and hinder the description of novel species. Clinical laboratories should maintain a high index of suspicion and employ confirmatory methods for precise isolates identification. Database curators and researchers must prioritize the validation and re-annotation of existing entries to correct longstanding errors. Such vigilance is vital for preserving the integrity of clinical diagnostics and scientific research.

Consistent with our findings, phylogenetic analysis based on single-copy orthologous genes showed that strains of the same species clustered within the same clade, with *P. huaxiensis* and *P. rettgeri* exhibiting close phylogenetic proximity. To improve species-level identification in clinical settings, a combination of phenotypic and genotypic approaches should be employed in the future. Additionally, misidentified *P. huaxiensis* genomes in GenBank should be revised to ensure taxonomic precision.

The prevalence of carbapenem-resistant *P. rettgeri* strains has increased in recent years, with carbapenemase genes frequently located on plasmids, transposons, and other mobile genetic elements (Zhang et al., 2022; Li et al., 2023; Shen et al., 2022). Several studies have reported *P. rettgeri* strains co-harboring *bla*_NDM_ and *bla*_OXA-10_. For instance, Shen et al. described the strain P138 co-harboring *bla*_NDM-1_, *bla*_VIM-1_, and *bla*_OXA-10_ genes (Piza-Buitrago et al., 2020), while Piza-Buitrago et al. reported two strains, GMR-RA257 and GMR-RA1153, producing NDM-1, VIM-2, and OXA-10 simultaneously (Carraro et al., 2015). Peng et al. identified an extensively drug-resistant (XDR) *Providencia rettgeri* strain co-harboring the *bla*_NDM-1_, *bla*_OXA-10_, and *tmexCD3-toprJ1b* gene clusters (Peng et al., 2023). Most of these isolates originated from China and exhibited similar MDR phenotypes, highlighting the need for surveillance of *Providencia* strains carrying both *bla*_NDM-1_ and *bla*_OXA-10_. Reports on carbapenemase-producing *P. huaxiensis* remain scarce, particularly those involving the co-production of NDM and OXA-10. In this study, we identified a novel SXT/R391 ICE carrying *bla*_NDM-1_ in a clinical *P. huaxiensis* strain PR66. This strain also harbored two copies of *bla*_OXA-10_, one located on the chromosome and the other on a plasmid.

The *bla*_NDM-1_ gene was located on a novel chromosomal ICE. BLAST analysis revealed no completely identical ICE sequences in existing databases, suggesting that ICE*Phu*Chn-PR66 is a new member of the MDR SXT/R391 ICE family. According to Burrus *et al*., ICEs encoding an integrase gene closely related to intSXT and integrated into the prfC locus are considered to be part of the SXT/R391 family. ICEs comprise core genes for integration/excision, conjugative transfer, and regulation, along with five hotspots (HS1–HS5) and four variable regions (VRI–IV) (Wozniak et al., 2009; Lei et al., 2016).

ICE*Phu*Chn-PR66 possesses a highly conserved backbone structure, including the integrase gene *int* and excisionase gene *xis* (recombination module); *oriT*, relaxase gene *traI*, T4CP gene *traD*, and T4SS genes *traLEKBVA*, *traC*, *trhF*, *traWUN*, *and traFHG* (conjugation module); and the *setCD* and *setR* genes (regulation module). These features may suggest that ICE*Phu*Chn-PR66 mediates the horizontal transfer of carbapenem resistance genes. Conjugation experiments confirmed the successful transfer of *bla*_NDM-1_ into *E. coli* C600. However, whether the *bla*_OXA-10_ gene could be transferred to recipient bacteria was not determined, and dissemination relationship between *bla*_NDM-1_ and *bla*_OXA-10_ remains unresolved.

We also observed that the genus *Proteus* is a major reservoir of SXT/R391 ICEs in the ICEberg database. Lei et al. first reported such ICEs in *P. mirabilis* isolates from China (Fu et al., 2022). Han et al. (2024) found that 25.2% of *P. mirabilis* isolates from farms were positive for SXT/R391 ICEs, exhibiting high overall drug resistance. Our findings showed that ICE*Phu*Chn-PR66 shares structural similarity with putative ICEs in *P. rettgeri* strains PROV002 (CP059345.1), PROV003 (CP096369.1) (Wang et al., 2023), and YK205 (CP090217.1) (Fu et al., 2022), suggesting a shared evolutionary origin and limited distribution within the genus *Providencia*.

Further analysis revealed that PR66 harbored two copies of the *bla*_OXA-10_ gene, one on the chromosome and one on plasmid pPR66.2. Both copies were located within highly similar integrons. The chromosomal copy was situated within the MDR region of ICE*Phu*Chn-PR66. BLAST alignment indicated that the integron was primarily plasmid-derived, including from the *P. rettgeri* plasmid pCHS4.1-1 (OL908906). These findings suggest that the integron with the *bla*_OXA-10_ gene on plasmid PR66.2 may have been transferred to the chromosome via ISCR elements.

This study has several limitations. First, WGS was not performed on all *P. rettgeri* strains in our laboratory to assess the regional prevalence of *P. huaxiensis*. Thus, the clinical and epidemiological burden of this emerging pathogen is likely underestimated. Second, while the conjugation experiment confirmed the transferability of the *bla*_NDM-1_ to recipient strains, the complete ICE into transconjugants was not verified by sequencing or PCR, and conjugation frequency was not calculated. Therefore, whether transfer of *bla*_NDM-1_ was mediated by the entire ICE or by other mobile genetic elements remains unclear. Third, while *bla*_OXA-10_ was found to be located on the same ICE as *bla*_NDM-1_, this study did not confirm whether it is co-transferred with the ICE, co-mobilized by the ICE, or transferred independently. Future studies should perform WGS on a larger collection of *Providencia* isolates to accurately determine the prevalence of *P. huaxiensis*. Additionally, it should be confirmed whether the ICE*Phu*Chn-PR66 carrying both *bla*_NDM-1_ and *bla*_OXA-10_ is transferred to the recipient, and the conjugation frequency should be determined under standardized conditions to investigate whether pPR66.2 can be co-transferred or mobilized by the ICE.

## 5 Conclusion

In conclusion, we found that *bla*_NDM-1_ is carried by a novel SXT/R391 family ICE named ICE*Phu*Chn-PR66 on the chromosome of *P. huaxiensis* PR66. The *bla*_NDM-1_ was transferable to *E. coli* C600 and may contribute to the dissemination of carbapenem resistance in *P. huaxiensis* and potentially other *Enterobacterales*. Our findings highlight the importance of ICEs in transmitting carbapenem resistance in *P. huaxiensis* and other bacterial species.

## Data Availability

The datasets presented in this study can be found in online repositories. The names of the repository/repositories and accession number(s) can be found in the article/Supplementary material.

## Footnotes

1
http://blast.ncbi.nlm.nih.gov/Blast.cgi

2
https://github.com/widdowquinn/pyani

3
http://www.drive5.com/muscle

4
http://www.microbesonline.org/fasttree/

## References

[ref1] Alikhan N. F. Petty N. K. Ben Zakour N. L. Beatson S. A. (2011). BLAST ring image generator (BRIG): simple prokaryote genome comparisons. BMC Genomics 12:402. doi: 10.1186/1471-2164-12-402, 21824423 PMC3163573

[ref2] Bortolaia V. Kaas R. S. Ruppe E. Roberts M. C. Schwarz S. Cattoir V. . (2020). ResFinder 4.0 for predictions of phenotypes from genotypes. J. Antimicrob. Chemother. 75, 3491–3500. doi: 10.1093/jac/dkaa345, 32780112 PMC7662176

[ref3] Botelho J. Schulenburg H. (2021). The role of integrative and conjugative elements in antibiotic resistance evolution. Trends Microbiol. 29, 8–18. doi: 10.1016/j.tim.2020.05.011, 32536522

[ref4] Carattoli A. Hasman H. (2020). PlasmidFinder and in silico pMLST: identification and typing of plasmid replicons in whole-genome sequencing (WGS). Methods Mol. Biol. 2075, 285–294. doi: 10.1007/978-1-4939-9877-7_20, 31584170

[ref5] Carraro N. Poulin D. Burrus V. (2015). Replication and active partition of integrative and conjugative elements (ICEs) of the SXT/R391 family: the line between ICEs and conjugative plasmids is getting thinner. PLoS Genet. 11:e1005298. doi: 10.1371/journal.pgen.1005298, 26061412 PMC4489591

[ref6] Castresana J. (2000). Selection of conserved blocks from multiple alignments for their use in phylogenetic analysis. Mol. Biol. Evol. 17, 540–552. doi: 10.1093/oxfordjournals.molbev.a026334, 10742046

[ref7] Cheng H. Concepcion G. T. Feng X. Zhang H. Li H. (2021). Haplotype-resolved de novo assembly using phased assembly graphs with hifiasm. Nat. Methods 18, 170–175. doi: 10.1038/s41592-020-01056-5, 33526886 PMC7961889

[ref8] Dong X. Jia H. Yu Y. Xiang Y. Zhang Y. (2024). Genomic revisitation and reclassification of the genus Providencia. mSphere 9:e0073123. doi: 10.1128/msphere.00731-23, 38412041 PMC10964429

[ref9] Dong X. Xiang Y. Shen P. Xiao Y. Zhang Y. (2025). Clinical emergence of Providencia zhejiangensis sp. nov. and Providencia xihuensis sp. nov.: genomic insights into antimicrobial resistance and geographical distribution. Int. J. Antimicrob. Agents 65:107484. doi: 10.1016/j.ijantimicag.2025.107484, 40023453

[ref10] Dong X. Xiang Y. Yang P. Wang S. Yan W. Yuan Y. . (2024). Novel Providencia xianensis sp. nov.: A multidrug-resistant species identified in clinical infections. Eur. J. Clin. Microbiol. Infect. Dis. 43, 1461–1467. doi: 10.1007/s10096-024-04821-y, 38714595 PMC11271419

[ref11] Dong X. Yu Y. Liu J. Cao D. Xiang Y. Bi K. . (2023). Whole-genome sequencing provides insights into a novel species: Providencia hangzhouensis associated with urinary tract infections. Microbiol. Spectrum 11:e0122723. doi: 10.1128/spectrum.01227-23, 37732781 PMC10581081

[ref12] Emms D. M. Kelly S. (2019). OrthoFinder: phylogenetic orthology inference for comparative genomics. Genome Biol. 20:238. doi: 10.1186/s13059-019-1832-y, 31727128 PMC6857279

[ref13] Fu S. Wang Q. Wang R. Zhang Y. Lan R. He F. . (2022). Horizontal transfer of antibiotic resistance genes within the bacterial communities in aquacultural environment. Sci. Total Environ. 820:153286. doi: 10.1016/j.scitotenv.2022.153286, 35074363

[ref14] Han Y. Gao Y.-F. Xu H. T. Li J. P. Li C. Song C. L. . (2024). Characterization and risk assessment of novel SXT/R391 integrative and conjugative elements with multidrug resistance in *Proteus mirabilis* isolated from China, 2018-2020. Microbiol. Spectrum 12:e0120923. doi: 10.1128/spectrum.01209-23, 38197656 PMC10871549

[ref15] Hickman-Brenner F. W. Farmer J. J. 3rd Steigerwalt A. G. Brenner D. (1983). J: *Providencia rustigianii*: a new species in the family Enterobacteriaceae formerly known as *Providencia alcalifaciens* biogroup 3. J. Clin. Microbiol. 17, 1057–1060. doi: 10.1128/jcm.17.6.1057-1060.1983, 6874899 PMC272801

[ref16] Hu Y. Feng Y. Zhang X. Zong Z. (2019). Providencia huaxiensis sp. nov., recovered from a human rectal swab. Int. J. Syst. Evol. Microbiol. 69, 2638–2643. doi: 10.1099/ijsem.0.003502, 31162027

[ref17] Hunt M. Silva N. D. Otto T. D. Parkhill J. Keane J. A. Harris S. R. (2015). Circlator: automated circularization of genome assemblies using long sequencing reads. Genome Biol. 16:294. doi: 10.1186/s13059-015-0849-0, 26714481 PMC4699355

[ref18] Hyatt D. Chen G.-L. Locascio P. F. Land M. L. Larimer F. W. Hauser L. J. (2010). Prodigal: prokaryotic gene recognition and translation initiation site identification. BMC Bioinformatics 11:119. doi: 10.1186/1471-2105-11-119, 20211023 PMC2848648

[ref19] Juneja P. Lazzaro B. P. (2009). *Providencia sneebia* sp. nov. and *Providencia burhodogranariea* sp. nov., isolated from wild *Drosophila melanogaster*. Int. J. Syst. Evol. Microbiol. 59, 1108–1111. doi: 10.1099/ijs.0.000117-0, 19406801

[ref20] Khunthongpan S. Sumpavapol P. Tanasupawat S. Benjakul S. H-Kittikun A. (2013). Providencia thailandensis sp. nov., isolated from seafood processing wastewater. J. Gen. Appl. Microbiol. 59, 185–190. doi: 10.2323/jgam.59.185, 23863288

[ref21] Ksentini I. Gharsallah H. Sahnoun M. Schuster C. Hamli Amri S. Gargouri R. . (2019). Providencia entomophila sp. nov., a new bacterial species associated with major olive pests in Tunisia. PLoS One 14:e0223943. doi: 10.1371/journal.pone.0223943, 31639141 PMC6805009

[ref22] Lei C. W. Zhang A. Y. Wang H. N. Liu B. H. Yang L. Q. Yang Y. Q. (2016). Characterization of SXT/R391 integrative and conjugative elements in *Proteus mirabilis* isolates from food-producing animals in China. Antimicrob. Agents Chemother. 60, 1935–1938. doi: 10.1128/AAC.02852-15, 26824957 PMC4775944

[ref23] Letunic I. Bork P. (2021). Interactive tree of life (iTOL) v5: an online tool for phylogenetic tree display and annotation. Nucleic Acids Res. 49, W293–W296. doi: 10.1093/nar/gkab301, 33885785 PMC8265157

[ref24] Li Z. Liao F. Ding Z. Chen S. Li D. (2022). Providencia manganoxydans sp. nov., a Mn(II)-oxidizing bacterium isolated from heavy metal contaminated soils in Hunan Province, China. Int. J. Syst. Evol. Microbiol. 72:005474. doi: 10.1099/ijsem.0.005474, 35930465

[ref25] Li Y. Shao K. Cai R. Liu Y. Liu X. Ni F. . (2023). Detection of NDM-1 and OXA-10 co-producing *Providencia rettgeri* clinical isolate. Infect Drug Resist 16, 5319–5328. doi: 10.2147/IDR.S418131, 37601562 PMC10439778

[ref26] Liu M. Li X. Xie Y. Bi D. Sun J. Li J. . (2019). ICEberg 2.0: an updated database of bacterial integrative and conjugative elements. Nucleic Acids Res. 47, D660–D665. doi: 10.1093/nar/gky1123, 30407568 PMC6323972

[ref27] Liu M. Yi N. Wang X. Wang R. (2023). Analysis of resistance genes of carbapenem-resistant *Providencia rettgeri* using whole genome sequencing. BMC Microbiol. 23:283. doi: 10.1186/s12866-023-03032-3, 37789331 PMC10546784

[ref28] Mohr O'Hara C. Steigerwalt A. G. Green D. McDowell M. Hill B. C. Brenner D. J. . (1999). M: isolation of *Providencia heimbachae* from human feces. J. Clin. Microbiol. 37, 3048–3050. doi: 10.1128/JCM.37.9.3048-3050.1999, 10449504 PMC85453

[ref29] Ondov B. D. Treangen T. J. Melsted P. Mallonee A. B. Bergman N. H. Koren S. . (2016). Mash: fast genome and metagenome distance estimation using MinHash. Genome Biol. 17:132. doi: 10.1186/s13059-016-0997-x, 27323842 PMC4915045

[ref30] Peng J. Xia Z. Zhang T. Zhao X. Chi L. Liu X. . (2023). Identification of tmexC3-tmexD3-toprJ1b in an XDR *Providencia rettgeri* clinical isolate co-producing NDM-1 and OXA-10 carbapenemases. J Glob Antimicrob Resist 34, 229–233. doi: 10.1016/j.jgar.2023.07.018, 37536658

[ref31] Piza-Buitrago A. Rincon V. Donato J. Saavedra S. Y. Duarte C. Morero J. . (2020). Genome-based characterization of two Colombian clinical *Providencia rettgeri* isolates co-harboring NDM-1, VIM-2, and other beta-lactamases. BMC Microbiol. 20:345. doi: 10.1186/s12866-020-02030-z, 33183231 PMC7664025

[ref32] Saavedra S. Y. Montilla-Escudero E. Wiesner M. Gonzalez M. N. Hidalgo A. M. Ovalle M. V. . (2022). First identification of the Bla(IMP-27) gene in a clinical isolate of *Providencia rettgeri* in Colombia. J Glob Antimicrob Resist 30, 428–430. doi: 10.1016/j.jgar.2022.05.005, 35569756

[ref33] Shaka M. Arias-Rojas A. Hrdina A. Frahm D. Iatsenko I. (2022). Lipopolysaccharide -mediated resistance to host antimicrobial peptides and hemocyte-derived reactive-oxygen species are the major *Providencia alcalifaciens* virulence factors in *Drosophila melanogaster*. PLoS Pathog. 18:e1010825. doi: 10.1371/journal.ppat.1010825, 36084158 PMC9491580

[ref34] Shen S. Huang X. Shi Q. Guo Y. Yang Y. Yin D. . (2022). Occurrence of NDM-1, VIM-1, and OXA-10 co-producing *Providencia rettgeri* clinical isolate in China. Front. Cell. Infect. Microbiol. 11:789646. doi: 10.3389/fcimb.2021.789646, 35047418 PMC8761753

[ref35] Shiroto K. Ishii Y. Kimura S. Alba J. Watanabe K. Matsushima Y. . (2005). Metallo-beta-lactamase IMP-1 in *Providencia rettgeri* from two different hospitals in Japan. J. Med. Microbiol. 54, 1065–1070. doi: 10.1099/jmm.0.46194-0, 16192438

[ref36] Singh V. K. Mangalam A. K. Dwivedi S. Naik S. (1998). Primer premier: program for design of degenerate primers from a protein sequence. BioTechniques 24, 318–319. doi: 10.2144/98242pf02, 9494736

[ref37] Somvanshi V. S. Lang E. Straubler B. Sproer C. Schumann P. Ganguly S. . (2006). *Providencia vermicola* sp. nov., isolated from infective juveniles of the entomopathogenic nematode Steinernema thermophilum. Int. J. Syst. Evol. Microbiol. 56, 629–633. doi: 10.1099/ijs.0.63973-0, 16514040

[ref38] Sullivan M. J. Petty N. K. Beatson S. A. (2011). Easyfig: a genome comparison visualizer. Bioinformatics 27, 1009–1010. doi: 10.1093/bioinformatics/btr039, 21278367 PMC3065679

[ref39] Walker B. J. Abeel T. Shea T. Priest M. Abouelliel A. Sakthikumar S. . (2014). Pilon: an integrated tool for comprehensive microbial variant detection and genome assembly improvement. PLoS One 9:e112963. doi: 10.1371/journal.pone.0112963, 25409509 PMC4237348

[ref40] Wang P. Li C. Yin Z. Jiang X. Li X. Mu X. . (2023). Genomic epidemiology and heterogeneity of Providencia and their Bla(NDM-1)-carrying plasmids. Emerg Microbes Infect 12:2275596. doi: 10.1080/22221751.2023.2275596, 37874004 PMC10796120

[ref41] Wozniak R. A. Fouts D. E. Spagnoletti M. Colombo M. M. Ceccarelli D. Garriss G. . (2009). Comparative ICE genomics: insights into the evolution of the SXT/R391 family of ICEs. PLoS Genet. 5:e1000786. doi: 10.1371/journal.pgen.1000786, 20041216 PMC2791158

[ref42] Xiang Y. Dong X. Ma L. Cao D. Li Y. Jiang X. . (2025). Taxonomic and phenotypic characterization of a novel Providencia species: Providencia lanzhouensis sp. nov. *microbiology*. Spectrum 13:e0054925. doi: 10.1128/spectrum.00549-25, 40631741 PMC12323597

[ref43] Yang W. Chen J. Yang F. Ji P. Shen S. Yin D. . (2024). Identification of a novel Providencia species showing multi-drug-resistant in three patients with hospital-acquired infection. Int. J. Antimicrob. Agents 64:107211. doi: 10.1016/j.ijantimicag.2024.107211, 38795927

[ref44] Zhang M. Yu Y. Wang Q. Chen R. Wang Y. Bai Y. . (2022). Conjugation of plasmid harboring Bla (NDM-1) in a clinical *Providencia rettgeri* strain through the formation of a fusion plasmid. Front. Microbiol. 13:1071385. doi: 10.3389/fmicb.2022.1071385, 36687647 PMC9845711

[ref45] Zurita J. Parra H. Gestal M. C. McDermott J. Barba P. (2015). First case of NDM-1-producing *Providencia rettgeri* in Ecuador. J Glob Antimicrob Resist 3, 302–303. doi: 10.1016/j.jgar.2015.07.003, 27842879

