# Supplementary material for: Identification of a novel SXT/R391 integrative and conjugative element harboring blaNDM-1 in a clinical Providencia huaxiensis isolate
Source: Front Microbiol. 2026 May 22;17:1818759. doi: 10.3389/fmicb.2026.1818759 (PMC13236907; doi:10.3389/fmicb.2026.1818759)
Supplement: Supplementary file 1 [file Table_1.DOCX]

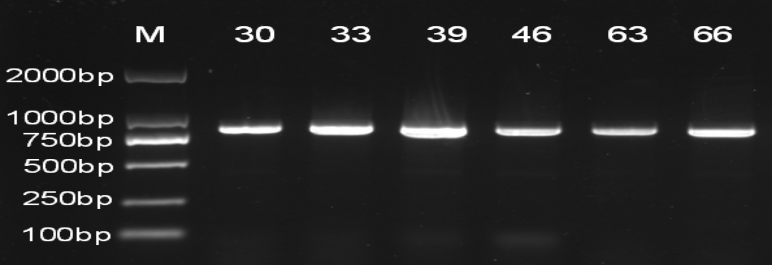


**Figure S1. Gel electrophoresis image for PCR detection of *bla*_NDM-1_ in clinical isolates.** The gel confirms the presence of the expected amplification product in the tested isolates and provides visual support for detection of *bla*_NDM-1_. PCR amplification was performed with *bla*_NDM_ primers yielding an expected 813-bp product. M, DL2000 DNA marker; lanes 1-6, six clinical isolates; lane 6, PR66. A band at approximately 813 bp indicates the presence of *bla*_NDM-1_ in the corresponding isolate.


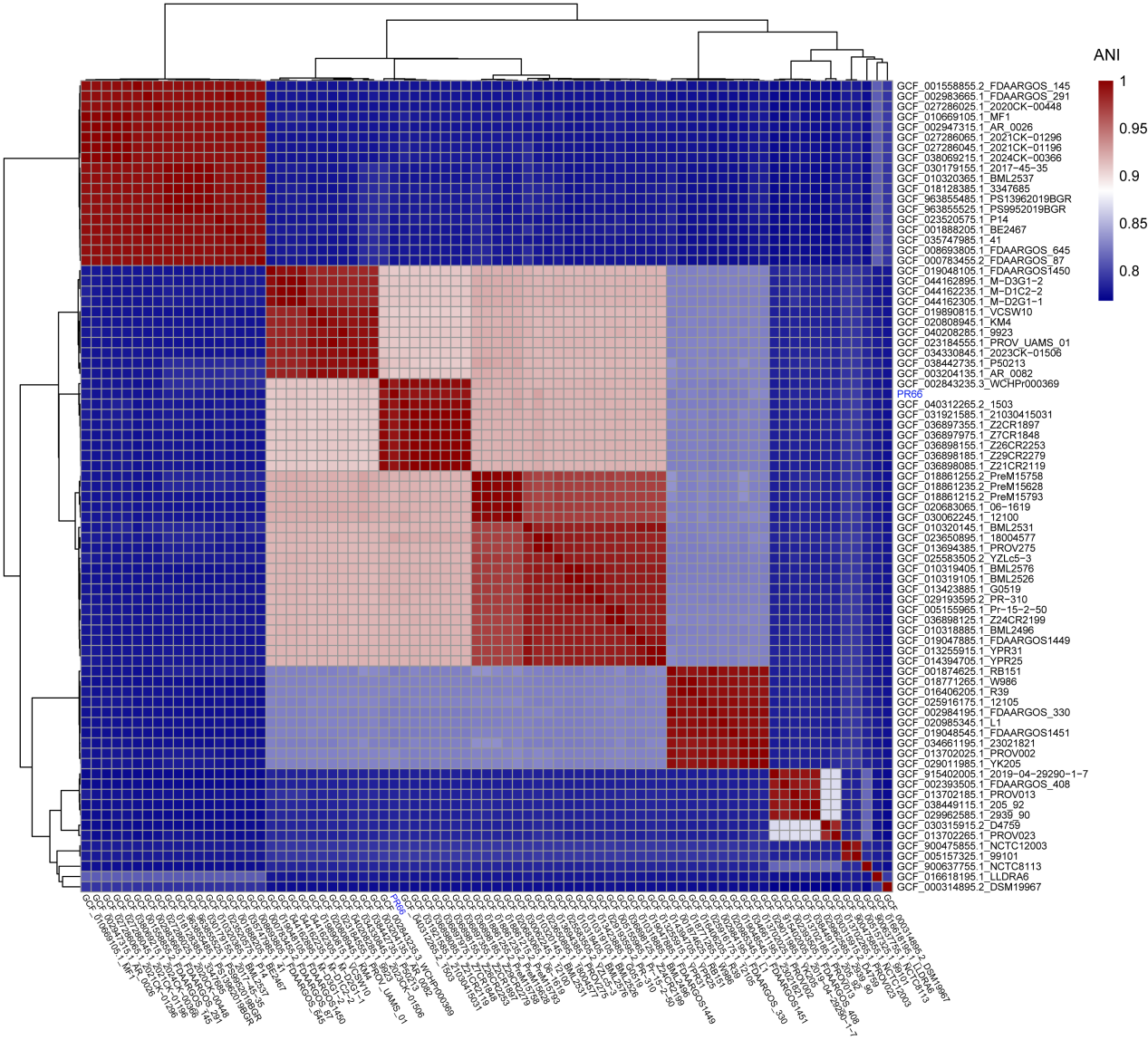


**Figure S2. Heatmap showing pairwise average nucleotide identity (ANI) values among 79 *Providencia* strains, including PR66 and 78 reference genomes downloaded from NCBI.** The full ANI matrix is provided in Supplementary Table 3.


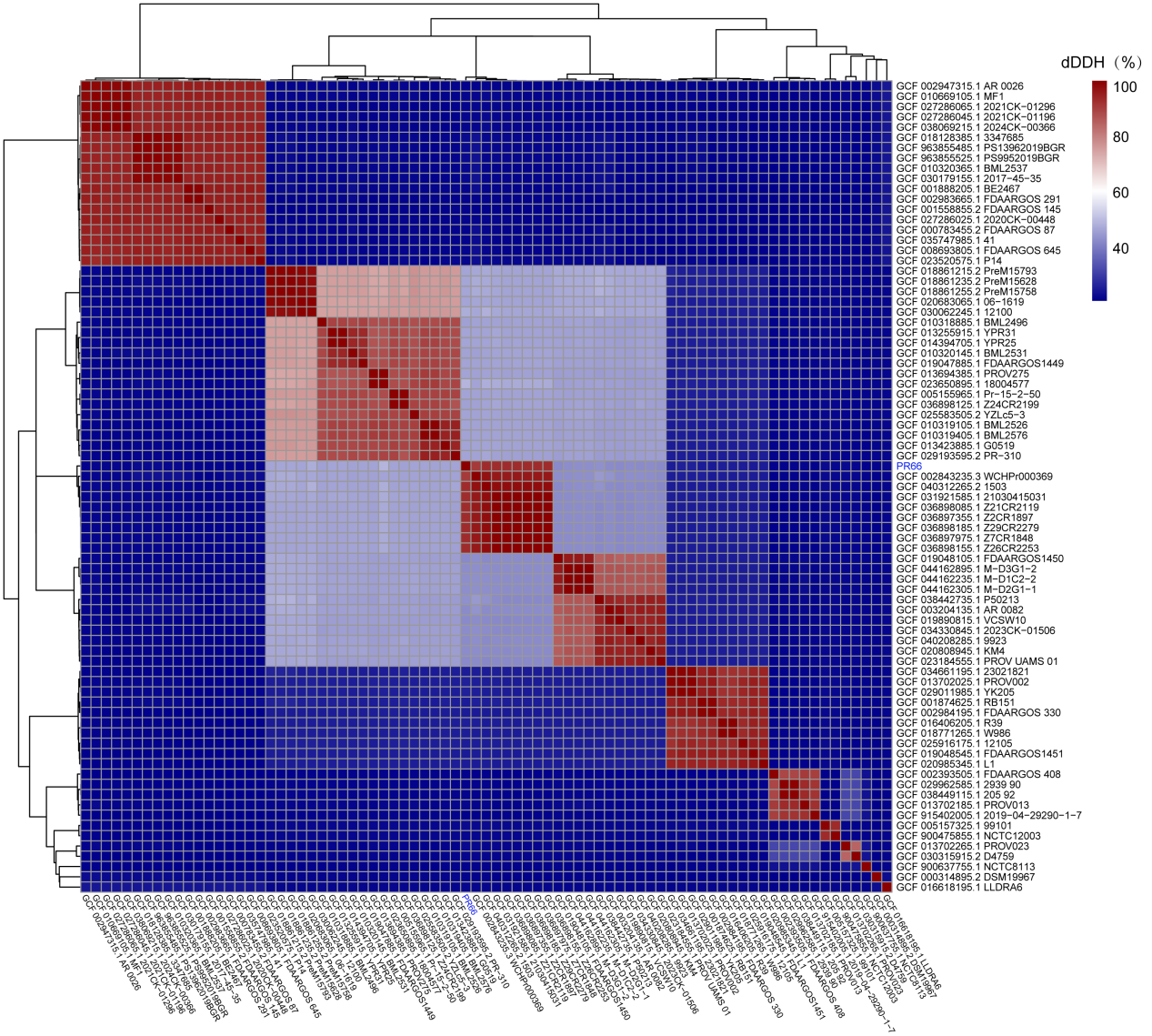


**Figure S3. Heatmap showing pairwise digital DNA-DNA hybridization (dDDH) values among 79 *Providencia* strains, including PR66 and 78 reference genomes.** The complete dDDH dataset is provided in Supplementary Table 4.


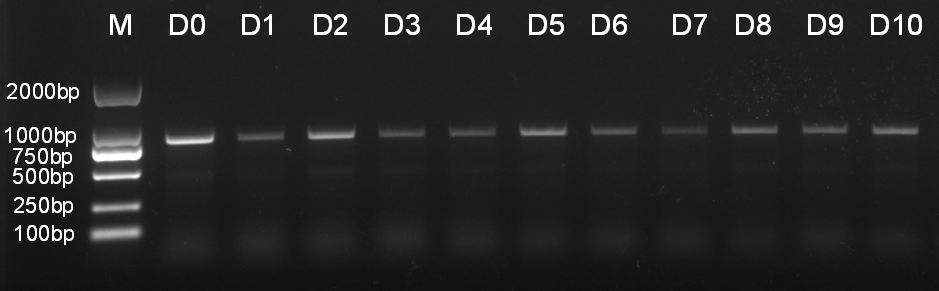


**Figure S4. Gel electrophoresis image for PCR detection of *bla*_NDM-1_ in PR66 transconjugants during serial passage.** The gel was included to assess persistence of *bla*_NDM-1_ during the 10-day stability experiment and to show whether the expected amplicon remained detectable over time. PCR was performed daily with *bla*_NDM_ primers yielding an expected 813-bp product. M, DL2000 DNA marker; lanes 1-11 correspond to days 0-10. Continued detection of an approximately 813-bp band across the serial-passage time points is consistent with maintenance of the transferred *bla*_NDM-1_-positive element in the transconjugant population.
